# Sensitivity of Neuroblastoma and Induced Neural Progenitor Cells to High-Intensity THz Radiation

**DOI:** 10.3390/ijms24076558

**Published:** 2023-03-31

**Authors:** Dmitry Sitnikov, Veronika Revkova, Inna Ilina, Rimma Shatalova, Pavel Komarov, Evgenia Struleva, Mikhail Konoplyannikov, Vladimir Kalsin, Vladimir Baklaushev

**Affiliations:** 1 Joint Institute for High Temperatures, Russian Academy of Sciences, 125412 Moscow, Russia; 2 Federal Research and Clinical Center of Specialized Medical Care and Medical Technologies, Federal Medical-Biological Agency of Russia, 119435 Moscow, Russia; 3 Engelhardt Institute of Molecular Biology, Russian Academy of Sciences, 119991 Moscow, Russia; 4 Center for Genetics and Life Sciences, Division of Genetics and Genetic Technologies, Sirius University of Science and Technology, 354340 Sochi, Russia; 5 Institute for Regenerative Medicine, Sechenov First Moscow State Medical University, 119435 Moscow, Russia; 6 Federal Center of Brain Research and Neurotechnologies, Federal Medical-Biological Agency, 117513 Moscow, Russia

**Keywords:** terahertz radiation, human neural progenitor cells, neuroblastoma, differentiation, proliferative activity, γH2AX, salinomycin, genotoxic effects

## Abstract

THz radiation induces a variety of processes in cells and has attracted the attention of researchers in recent decades. Here, data on the effects of high-intensity terahertz (THz) radiation on human directly reprogrammed neural progenitor cells (drNPCs) and on neuroblastoma cells (SK-N-BE (2)) were obtained for the first time. The results demonstrated that the exposure of non-tumor and tumor cells to broadband (0.1–3 THz) THz pulses with the intensity of 21 GW/cm^2^ and the electric field strength of 2.8 MV/cm for 30 min induced neither a noticeable genotoxic effect nor a statistically significant change in the proliferative activity and cell differentiation. It was also shown that the combined effect of THz radiation and salinomycin, a promising antitumor agent, on neuroblastoma cells did not enhance the genotoxic effect of this antibiotic. However, further studies involving chemotherapy drugs and other exposure parameters are warranted to introduce this new concept into anti-tumor clinical practice and to enhance the efficacy of the existing approaches.

## 1. Introduction

The terahertz (THz) spectral region of electromagnetic radiation is commonly referred to as the frequency range between 0.1 and 10 THz, wavelengths between 30 μm and 3 mm, and photon energies from 0.41 to 41 meV. The recent advances in THz generation and detection techniques have led to widespread applications covering medical imaging, diagnostics, and therapy (see reviews [1,2,3,4]). These applications include the diagnosis of malignant and benign neoplasms [5], intraoperative neurodiagnostics [6], determination of the hydration level [7] and viability of tissue, as well as regulation of the expression of genes associated with systemic inflammatory diseases/cancer [8], and the application of *Escherichia coli* bacteria as biosensors for THz radiation [9]. Despite the low energy and non-ionizing nature of THz photons, one should be aware of the fundamental biological effects of THz radiation when developing THz-based diagnostic and therapeutic technologies. It has been shown (see reviews, e.g., [10,11,12,13]) that THz radiation can have both thermal and non-thermal effects on biological objects.

A number of studies demonstrate that the nervous system is sensitive to non-ionizing electromagnetic radiation [14,15,16]. However, only a few studies deal with the effects of THz radiation on the nervous system cells. For instance, Zhao et al., showed that THz radiation inhibited the proliferation of progenitors of mouse oligodendrocytes, but stimulated their differentiation in the mature forms with the following myelination of fibers [17]. An extensive study by Tan Sheng Zhi et al., showed that different neuron types, in both normal and tumor rodent cell lines, were distinct in their sensitivity to THz radiation [18]. In particular, THz irradiation of laboratory animals with a power of 10–50 mW (0.16–0.17 THz) and exposure of 6–60 min did not result in altered morphology or growth of neurites into primary neurons and neuron-like cells. However, changes in the neurotransmitter subcellular distribution were observed: The THz radiation increased the permeability of neuron-like cells’ membranes and provided regulation of various neurotransmitters, such as Glu, Gly, and Ala. Interestingly the found effects correlated with the increase in power or exposure to the THz irradiation.

The frequencies of terahertz waves correspond to conformational vibrations of biomolecules, which means such waves can excite them to higher vibrational and rotational energetic levels, result in changes in their molecular conformation, and affect interactions between biological macromolecules [19]. All of this can lead to a change in gene expression [20] and an increase in the level of phosphorylated histones H2AX [8], and can serve as indicators of cell sensitivity. The formation of the foci of γH2AX phosphorylation upon the THz exposure may indicate a genotoxic effect. An increase in the level of the p53 protein, which is a tumor suppressor and affects the cell cycle, was observed earlier [21]. In our previous study [22,23], we showed that a high peak power of THz radiation can increase the number of the γH2AX foci in human skin fibroblasts. In addition, the number of the γH2AX foci is detected even after a short exposure (30 min) and increases with time.

The combined effects of electromagnetic radiation (both ionizing and non-ionizing) and drugs are well-known in oncological practice [24,25]. Irradiation, as well as the action of anti-tumor drugs, may cause DNA damage in tumor cells, followed by their apoptosis. The combined therapy allows for reducing the tumor’s resistance to chemotherapy and decreasing the adverse effects on the healthy tissues. In 2009, Gupta et al., conducted a study in which they found that salinomycin selectively eliminated cancer stem cells (CSCs) separated from a human breast cancer tumor [26]. It was also demonstrated that salinomycin caused apoptosis of cancer cells, which were resistant to other traditional chemotherapy drugs [27]. Salinomycin appeared efficient in inhibiting tumor cells of the pancreas [28], in colorectal cancer [29], chronic lymphocytic leukemia [30], glioblastoma [31], melanoma [32], etc. Recently, Wang et al., demonstrated that salinomycin suppresses the growth of human neuroblastoma cells, binding to the nucleolin target protein, which is a critical regulator of the neuroblastoma CSCs’ activity [33]. Neuroblastoma is the most frequently diagnosed solid extracranial malignant tumor in the pediatric population, and the mortality from neuroblastoma represents 15% of early childhood cancer mortality. The treatment options are limited for as many as 60% of patients. Since THz radiation promotes the demethylation of DNA [34], it may play the role of an epigenetic inhibitor for tumors.

The purpose of this study was to uncover the THz radiation effects on the proliferation and differentiation of directly reprogrammed human neural progenitor cells (drNPCs), as well as on human neuroblastoma cells (SK-N-BE (2)). The irradiation of the cells was conducted with the highest available pulse energy of 10 µJ, corresponding to an intensity of 21 GW/cm^2^ and exceeding the values in the other mentioned studies. In addition, the genotoxic effect of strong THz radiation alone on human drNPCs (relatively normal neuron-like cells) and neuroblastoma cells, as well as in combination with salinomycin, a promising anti-tumor agent, on neuroblastoma cells, were studied, based on the analysis of the number of foci of histone H2AX (γH2AX) phosphorylation.

## 2. Results

### 2.1. Assessment of Spontaneous Differentiation of Neural Progenitor Cells (drNPCs) and Human Neuroblastoma Cells (SK-N-BE (2))

CSCs represent a cell population inside a tumor, which is capable of self-renewal and differentiation, thus increasing the survival of the tumor population after radiotherapy or chemotherapy. The modern approaches to the treatment of oncological diseases include strategies that not only result in the death of tumor cells but also allow for reversing metastasis and inducing differentiation of CSCs.

In the framework of this work, we studied the effect of THz radiation on the differentiation of non-tumor and tumor neural cells. The cultures of human drNPCs and SK-N-BE (2) neuroblastoma cells were exposed to THz radiation with the preset parameters for 30 min.

**Figure 1:**
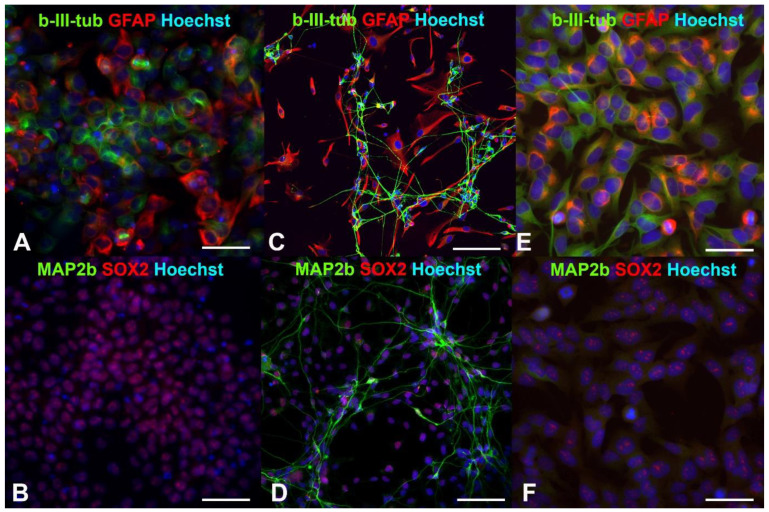
Fluorescent immunocytochemistry of human neural progenitor cells (drNPCs) and human neuroblastoma cells (SK-N-BE (2)): (**A**,**B**) Phenotype of human drNPC in proliferation medium; (**C**,**D**) neuronal and glial differentiation of human drNPCs induced by growth factors (positive control); (**E**,**F**) immunophenotype of human neuroblastoma cells (SK-N-BE (2)). Cells were stained with cocktails of primary antibodies (mouse monoclonal + rabbit polyclonal), followed by staining a cocktail of goat anti-mouse and goat anti-rabbit secondary antibodies with Alexa Fluor 488 (green channel) and Alexa Fluor 633 (red channel), respectively. The cell nuclei were stained with Hoechst (blue channel) in all the images. Scale bar 100 µm for (**A**–**D**) and 50 µm for (**E**,**F**).

Earlier, we showed that human drNPCs efficiently expressed the pluripotency markers, such as SOX2 and nestin, and co-expressed neuronal β-III-tubulin and glial intermediate filament protein—GFAP [35,36] (Figure 1A,B). As a result of the induced differentiation into mature neurons, the SOX2 disappears, and the expression of β-III-tubulin and GFAP is distributed between the neurons and glial cells, respectively (Figure 1C). One can observe MAP2b-positive mature neurons appearing later (Figure 1D).

**Figure 2:**
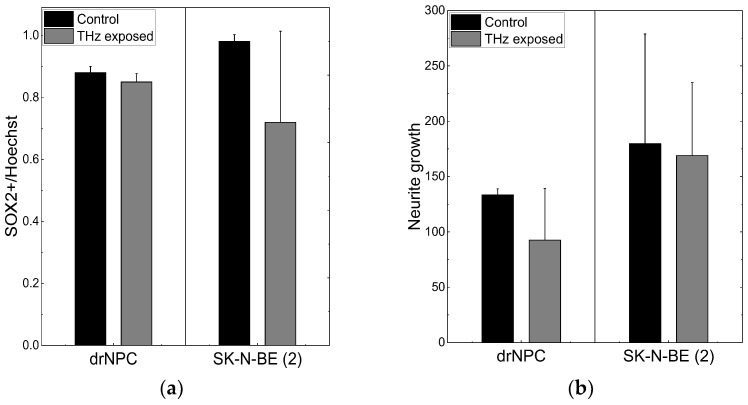
THz radiation effect on differentiation of drNPC and SK-N-BE (2): (**a**) The ratio of the number of SOX2-positive cells to the total number of cells in the experimental groups and in the parallel control groups; (**b**) the growth of neurites (µm) based on the analysis of β-III-tubulin expression in the irradiated cells and in the parallel control groups.

Similar to human neural progenitor cells, human neuroblastoma cells are characterized by a high content of SOX2+ cells and co-expression of the β-III-tubulin and GFAP (Figure 1E,F). After irradiation with the THz pulses for 30 min, more than 66% of the neuroblastoma cells maintained their SOX2 expression (Figure 2a), and no mature MAP2b+ neurons were observed. Thus, the THz irradiation did not result in the appearance of cells morphologically and phenotypically similar to mature neurons and/or astrocytes, as it occurred in the case of induced differentiation of neural progenitor cells (Figure 1C,D).

No significant reduction of the number of SOX2-positive stem-like cells due to the THz irradiation was observed in either the non-tumor or tumor cell cultures (*p* < 0.05), and no changes in the morphology cells were found, and no mature MAP2b+ appeared. In addition, no signs of differentiation were observed in the cultures of the neural progenitor cells and neuroblastoma cells after the THz irradiation (Figure 2b). Thus, no effect of THz radiation on the differentiation of drNPC and human neuroblastoma cells (SK-N-BE (2)) was revealed.

### 2.2. Studies of the Cells’ Proliferative Activity and Foci of Histone H2AX Phosphorylation

Here, we estimated the genotoxic effect of THz radiation using a quantitative analysis of the foci of γH2AX phosphorylation. It was found that phosphorylation of histone H2AX at serine 139 was one of the early stages of the cell response to double-strand DNA breaks [37]. The genotoxic stress leads to an accumulation of γH2AX in the regions of DNA damage as soon as several minutes after the damage, followed by its spreading over millions of base pairs from the initial damaged region. The appearance of the γH2AX foci associated with double-strand DNA breaks may also be related to cell death or neoplastic transformation [38].

**Figure 3:**
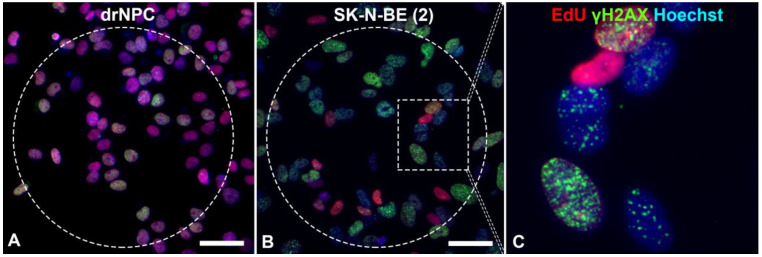
Immunofluorescent analysis of the γH2AX foci in: (**A**) drNPCs and (**B**,**C**) SK-N-BE (2) cells. The THz-irradiated region is marked with a dashed line. The γH2AX foci (green) are found both in cells in the synthesis phase of the cell cycle (EdU, red) and those in the post-synthesis phase. The cell nuclei are stained with Hoechst (blue) in all the images. Circle denotes the area of THz exposure. Square denotes the magnified region shown in section (**C**). Scale bar 100 µm.

The level of histone H2AX phosphorylation may remain normal in aging cells and in cells undergoing the natural cell death pathway—apoptosis. During the S phase of the cell cycle, active replication takes place, which requires the decondensed state of chromatin and leads to its increased amount, which proportionally increases the number of the γH2AX foci [39]. Therefore, we performed an estimation of the number of foci of histone phosphorylation in cells, which were not in the synthesis phase of the cell cycle. To identify cells in the S phase of the cell cycle, 5-ethynyl-2′-deoxyuridine (EdU) was applied to the DNA structure. The analyses revealed that most of the human neuronal stem-like cells were in the S phase of the cell cycle, which reflected a high proliferative activity of these cell types (Figure 3).

**Figure 4:**
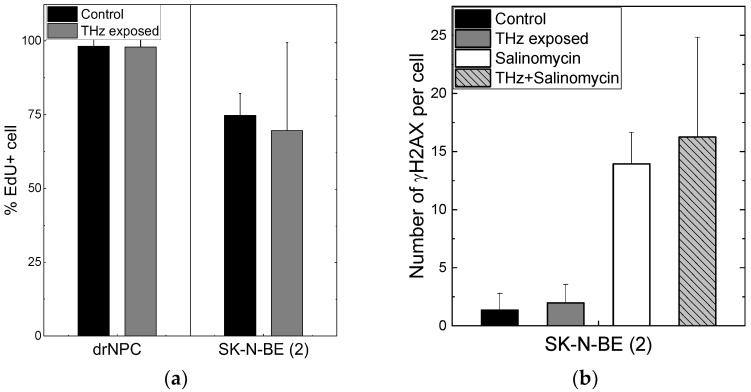
Proliferative activity of neuronal cells and formation of the foci of histone H2AX phosphorylation: (**a**) The number of cells in the synthesis phase of the cell cycle, detected through the EdU incorporated in cell DNA; (**b**) the number of γH2AX per cell in the human neuroblastoma line (SK-N-BE(2)).

The number of human drNPC in the S phase was essentially higher than that of SK-N-BE (2), both in the parallel control group (~75%), and in the experimental group (~70%) after the THz irradiation (Figure 4a). However, while the proliferation of human drNPC after THz remains stable, the proliferation of neuroblastoma cells (SK-N-BE (2)) after irradiation decreases (~5%), though this decrease is not statistically significant. Thus, one may conclude that strong non-ionizing terahertz radiation does not affect the proliferative activity of either non-tumor or tumor cells.

No differences were observed in the numbers of the foci *N*_f/cell_ of histone H2AX phosphorylation per cell in the experimental (*N*_f/cell_ = 0 ± 0) and control (*N*_f/cell_ = 0 ± 0) groups of human drNPC. The histone number in neuroblastoma cells (SK-N-BE (2)) after 30 min of irradiation (*N*_f/cell_ = 1.97 ± 1.60) exceeded the control value (*N*_f/cell_ = 1.36 ± 1.44) by approximately 1.5 times; however, this difference was not significant statistically (Figure 4b). The obtained data testify that the THz exposure of 30 min does not change the number of the foci of histone H2AX phosphorylation either for neuroblastoma cells (SK-N-BE (2)) or neural stem cells (drNPCs).

We showed earlier that the salinomycin solution concentration most efficient against tumor cells is 10–20 µM [40]. In this study, we used the concentration of 10 µM to test the hypothesis about the efficacy of a combined action of salinomycin and THz radiation. Since salinomycin is highly neurotoxic, which leads to the death of non-cancerous neural progenitor cells, we did not include them in this study. As shown above, the number of the foci of histone H2AX phosphorylation is the measure of the genotoxic effect. To estimate the combined effect of salinomycin and THz radiation, the number of the γH2AX foci was analyzed (Figure 4b). The difference between the numbers of the γH2AX foci per cell for SK-N-BE (2) in the experimental group and in the parallel control group is not statistically significant (Figure 4b). The addition of salinomycin to neuroblastoma cells in the concentration of 10 µM resulted in a fivefold increase in the number of foci vs. that for the parallel control group. The combined action of the THz radiation and salinomycin manifested itself in a certain increase in the number of foci as compared to that for salinomycin alone; however, these changes were not statistically significant. Thus, the combined treatment with THz radiation and salinomycin does not boost the genotoxic effect of the antibiotic.

## 3. Discussion

It is a known fact that, upon addition of the factors of neuronal and glial differentiation, directly reprogrammed human neural progenitor cells (drNPCs) demonstrate a decrease in the number of SOX2-positive cells, as well as a reduction of the number of GFAP+ and β-III-tubulin+ co-expressing cells, which indicates the loss of the cell culture’s stem properties. In addition, the appearance of MAP2b-positive cells with long sprouts, similar to dendrites, confirms the human drNPCs’ differentiation into mature neurons. However, the attempt to cause differentiation initiated by THz radiation did not lead to changes in the morphology of the cell lines or the expression of specific markers. The tumor line, SK-N-BE (2), cells and the human neural progenitor cells (drNPC) predominantly maintained their multi-potency after irradiation, as testified by a high fraction of SOX2-positive cells and by the absent expression of the marker for mature neurons, MAP2b. At the same time, the co-expression of GFAP and β-III-tubulin remained intact. Thus, the action of strong broadband non-ionizing radiation (0.2–3.0 THz) with a peak intensity of ~21 GW/cm^2^ and electric field strength of ~2.8 MV/cm did not initiate the differentiation of cells towards mature neurons or glia, either in human neuroblastoma cells (SK-N-BE (2)) or in human neural progenitor cells (drNPCs). The obtained results agree with the literature data, which report that a 1 h exposure of human primary keratinocytes (NHKs) to radiation of 1–3 THz did not affect the cells’ capability of differentiation [41]. In contrast to the data obtained, a recent study [17] demonstrated the ability of THz radiation (source of continuous radiation at 3.1 THz, 70 mW/cm^2^) to inhibit the proliferation of oligodendrocyte precursor cells and promote their differentiation. The myelination process was enhanced after the THz exposure. These results suggest that THz irradiation can modulate the functions of different neuronal cells with different sensitivities to THz radiation (mouse primary cortical neurons and oligodendrocytes). Another possible conclusion is that monochromatic radiation at a given frequency could be more efficient than broadband cell exposure. In another study [42], NHKs underwent treatment with 0.14 THz radiation, with the duration and intensity varying from 10 min to 24 h and 24 to 62 mW/cm^2^, respectively. No adverse effects on differentiation were detected by the test, which was based on the formation of cornified envelopes.

The change in the number of the foci of histone H2AX (γH2AX) phosphorylation is a sensitive marker for double-strand DNA breaks and is widely used in genotoxicity studies. At the same time, it should be noted that the spontaneous level of the γH2AX foci in cell lines may be variable. Apoptotic cell death and replicative aging are also associated with the accumulation of double-strand breaks and, hence, with the formation of the γH2AX foci. In addition, the increased number of the γH2AX foci may not only indicate double-strand DNA breaks but also result from hyperthermia or alterations of the chromatin state. Spontaneously high levels of the foci may emerge in cells without exogenic stress.

A large number of the γH2AX foci (up to 50 per nucleus) was also observed in cells of proliferating cultures of certain cell lines derived from human solid tumors. Cell lines expressing mutant p53 or those without p53 expression (such as SK-N-BE (2)) showed significantly higher numbers of the γH2AX foci [43]. The action of THz radiation does not significantly increase the number of the induced foci of phosphorylation in neuroblastoma cells (SK-N-BE (2)). We also did not observe any genotoxic effect of THz radiation on human neural progenitor cells (drNPCs). In total, the obtained results agree with the data of a similarly designed experiment, in which human embryonic stem cells underwent exposure to narrowband 2.3 THz waves at the mean power density of 1.4 W/cm^2^ and a scrupulously controlled temperature [44]. No genotoxic effect was also found in our previous study of neuroblastoma and glioblastoma exposed to pulses of THz radiation with the same parameters [45]. One may assume that the absence of effects is related to the short duration of the irradiation or the used cultures. However, in another study on a model of human skin cells with an exposure time of up to 24 h, the genotoxic effect also was not noted [46]. Nevertheless, in the future, it would be beneficial to expand the utilized parameters—cell cultures and time of irradiation.

The combined treatment with salinomycin and THz radiation also did not increase the number of the phosphorylated γH2AX foci, which did not statistically differ from that for the salinomycin treatment alone. This finding indicates the absence of a sensitizing effect and may be associated with the short duration of irradiation or with the sufficiently strong negative effect of the antibiotic itself on neuroblastoma cells. Our results are in agreement with the literature data on salinomycin efficiently killing cancer stem cells. Salinomycin is a polyether ionophore antibiotic extracted from Streptomyces albus [26,47,48,49]. Salinomycin suppresses CSCs through mechanisms that are still understudied. In particular, salinomycin is known to promote iron accumulation and sequestration in cell lysosomes [48]. In response to the exhaustion of the iron pool in the cytoplasm, cells launch the degradation of ferritin in lysosomes, which results in further iron accumulation in these organelles. Iron-mediated production of reactive oxygen species leads to the enhanced permeability of the lysosomal membrane and the activation of the cell death pathway corresponding to ferroptosis. In addition, Liu et al., showed that salinomycin was able to suppress CSCs in the liver and induce their differentiation via the Wnt/β-catenin signal pathway [49]. Salinomycin also suppresses the expression of the ATP-binding cassette transporter in multidrug-resistant cells and interferes with key signaling pathways involved in cell survival and cancer progression (PI3K/Akt, Wnt/β-catenin, Hedgehog, Notch) [47,50].

We showed earlier that salinomycin-based preparations (salinomycin-loaded mesoporous silicon nanoparticles) demonstrated a notable cytotoxic effect on human glioblastoma cells, and MCF-7 and MCF-7/MDR1 breast cancer cells. The experiments in vivo demonstrated that the growth of Lewis lung carcinoma was essentially suppressed after intraperitoneal administration of the salinomycin-loaded nanocomposite [40]. Those results are in good agreement with the data on salinomycin treatment of melanoma in vitro and in vivo. In particular, salinomycin-induced autophagic cell death in six melanoma cell lines was observed by Liu et al. [51]. Another study by Zhou et al., showed salinomycin’s ability to inhibit the growth and survival of uveal melanoma cells, both in vitro and in vivo, in a xenograft murine model. The CSCs were essentially eliminated, and hepatic metastasis was inhibited in a murine model of liver-metastatic uveal melanoma [32].

Terahertz radiation has significant potential in medical diagnosis and treatment due to its frequency range matching the characteristic energy of the biomolecular motion. Successful examples of THz diagnostics include skin, oral, gastric, breast, cervical, prostate, and brain cancers (see reviews [1,4,52]). High power sources of THz radiation enhance the depth of penetration into water-containing biological tissues and may improve anti-tumor treatment through the demethylation of malignant DNA at a specific frequency. Intense THz pulses were shown to induce concerted positive changes in the expression of genes related to inflammatory skin diseases and cancers, which gives rise to potential medical applications for this technique [8]. The above considerations allow us to assume that the combined treatment of various tumor cells with salinomycin and THz radiation may be more efficient that their treatment with salinomycin alone.

However, for the case of the neural cells we used, one may conclude that high-intensity broadband terahertz radiation does not induce a noticeable genotoxic effect, statistically significant alteration of the proliferative activity, or differentiation of cells when applied to non-tumor or tumor cells. No sensitizing effect is observed upon the combined action of THz radiation and salinomycin. It would possibly be reasonable to expand the experimental parameters in future studies. In this study, the effects of high-intensity pulses of THz radiation were tracked for a relatively short exposure time of 30 min, while the long-term effects of THz radiation on these cells remain unknown. Another limitation to be addressed in our future studies is related to the intensity of the THz radiation. Upgrading the experimental scheme will enable us to boost the radiation intensity and to study the effects reported here in the coordinates of “time–intensity”. The obtained data will gain new insights into the mechanism of electromagnetic field interference with the processes of cell differentiation. They may also be utilized in finding the optimal exposure parameters for the potential medical applications of THz radiation.

## 4. Materials and Methods

### 4.1. Description of the THz Setup for Irradiation of Cells

**Figure 5:**
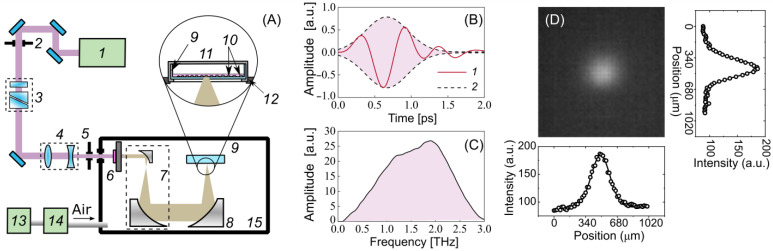
Experimental setup for cell irradiation. (**A**) Schematic: 1—femtosecond laser, 2, 5—iris diaphragm, 3—polarization attenuator, 4—lens telescope, 6—OH1 crystal with a filter, 7—mirror telescope, 8—focusing mirror, 9—Petri dish, 10—cells, 11—heated plate and cover, 12—3-axis linear stage, 13—adsorption desiccant, 14—air compressor, 15—dry box. (**B**) Waveform of the THz pulse: 1—THz waveform, 2—Gaussian envelope; (**C**) spectrum of the THz pulse; (**D**) spatial distribution of THz radiation.

Optical rectification (OR) is one of the most efficient techniques for converting laser radiation into terahertz radiation, with a conversion of up to 3% [53]. An OH1 [(2-(3-(4-hydroxystyryl)-5,5-dimethylcyclo-hex-2-enylidene) malononitrile] organic crystal was used for generating pulses of THz radiation. Figure 5A demonstrates an experimental setup for the cell exposure to high-intensity THz pulses, which is described in detail in [54,55]. The crystal was pumped by the radiation of a Cr:forsterite femtosecond laser system (pulse duration $\tau_{L}$ = 100 fs, wavelength *λ* = 1240 nm, energy $E_{L}$ = 1.1 ± 0.05 mJ, pulse repetition rate $f_{\mathrm{rep}}$ = 100 Hz) [56]. A polarization attenuator consisting of a half-wave plate and a Glan–Thomson prism was used to attenuate the energy of the laser pulses. A Galilean telescope (2:1 ratio) (Thorlabs Inc., Newton, NJ, USA) was designed to obtain the laser energy density required for the optimal conversion efficiency in the OH1 crystal. It has been shown that the THz yield tends to saturate as the laser fluence grows in OR schemes [57,58,59]. A broadband 0.2–3 THz subpicosecond pulse was generated, the waveform and the spectrum of which are presented in Figure 5B,C. The laser radiation was subsequently cut off by a THz filter (LPF8.8-47, Tidex LLC, London, UK) with a 70% transmission installed immediately after the THz crystal. A reflecting telescope consisting of two off-axis parabolic mirrors (Thorlabs Inc., Newton, NJ, USA) expanded the beam diameter (1:10 ratio) for better subsequent focusing. To achieve the maximum intensity and electric field strength of the THz pulses for the cell exposure, the THz radiation was focused using a similar gold off-axis parabolic mirror (Thorlabs Inc.). An uncooled micro-bolometer camera (Rigi, Swiss THz, Buchegg, Switzerland) was used to estimate the spatial energy distribution of the THz radiation in the beam waist (Figure 5D). The radiation was concentrated in a spot with a diameter of $d_{1/e}$ = 480 μm at the 1/e level, close to the diffraction limit.

A high peak power allows the radiation to penetrate through the aqueous medium, which has strong absorption in the THz frequency range. To minimize the losses associated with the absorption of the THz radiation by water vapor in the air, the THz elements of the experimental scheme were assembled in a box filled with dry air, with a relative humidity of 2–3% at room temperature. A 35 mm ibidi Petri dish (#80466, ibidi) with a cell monolayer was placed in a heating system (ibidi) mounted atop a three-axis linear stage. The cells were irradiated by a focused beam of THz radiation through the bottom of the plastic Petri dish. The maximum energy of a THz pulse of 10 µJ reaching the cells made it possible to achieve the intensity and electric field strength in the THz beam waist of up to 21 GW/cm^2^ and 2.8 MV/cm (calculation details are given elsewhere [23,60]), respectively.

### 4.2. Cell Culture

Human neural progenitor cells (drNPCs) were provided by New World Laboratories, Inc. (Laval, QC, Canada) [61]. The cell cultures were kept in Petri dishes covered with Matrigel (1:100, BD, Franklin Lakes, NJ, Canada), in the medium of the NeuroCult-XF Proliferation Kit (StemCell Technologies; Burnaby, BC, Canada) with the addition of 1% B27 (Gibco, Waltham, MA, USA), epidermal growth factor (EGF) [20 ng/mL] (Gibco, Waltham, MA, USA), and fibroblast growth factor 2 (FGF-2) [20 ng/mL] (Gibco, Waltham, MA, USA) in the conditions of a multi-gas incubator with 5% CO_2_ and 5% O_2_ at 37 °C. The culture medium was replaced every two days. When needed, the cells were passaged at the ratio of 1:5 at 80% confluency. To induce the differentiation of drNPCs, the standard culture medium was replaced with the NeuroCult-NS-A Medium (#05750, #05753 StemCell Technologies; Burnaby, BC, Canada), with the addition of 1% B27 (#17504044, Gibco, Waltham, MA, USA), 1% CultureOne (#A3320201, Gibco, Waltham, MA, USA), 40 ng/mL of BDNF (No. 130-096-286, Miltenyi Biotec, Bergisch Gladbach, Germany), and 20 ng/mL of GDNF (No. 130-098-449, Miltenyi Biotec, Bergisch Gladbach, Germany). The medium was replaced every 3 days. The cells were cultured for no less than 14 days.

Human neuroblastoma cells of the SK-N-BE (2) line were seeded on Petri dishes with a density of 1.5 × 10^5^ cells/cm^2^. The culture medium was Dulbecco’s Modified Eagle Medium mixed with Ham’s medium (DMEM/F12, Gibco, Waltham, MA, USA), with the addition of 10% fetal bovine serum (Gibco, Waltham, MA, USA), 2 mM of L-glutamine (GlutaMAX, (Gibco), and an antibiotic-antimycotic solution (1×; Gibco, Waltham, MA, USA). The cells were cultivated in the conditions of an incubator in Petri dishes at 37 °C and 5% CO_2_.

### 4.3. Preparation of Samples for Irradiation

One day before the experiment, the cells were placed into culture dishes with a polymer bottom, transparent for radiation with the THz region of frequencies, and special silicone inserts (ibidi, #80466). The cell line was treated with the Accutase solution (#07920 StemCell Technologies, Burnaby, BC, Canada); the total number of cells was calculated; and the cells were transferred to culture dishes at the concentration of 5 × 10^4^ cells/cm^2^. To maintain the acid–base balance of the medium during irradiation, a 15 mM HEPES (Gibco) solution was preliminarily added to the culture medium. The prepared dishes were divided into two groups for each line: the experimental group (which underwent the THz radiation pulses) and the parallel control group (which was kept in the same conditions, but without exposure to the THz radiation).

### 4.4. Irradiation of Samples

Titova et al., showed that a 10 min-long exposure of artificial human skin tissues to intense pulses of THz radiation was enough to induce alteration of the gene expression [8] and increase the γH2AX levels [21]. Our previous investigations [23] demonstrated that the exposure of human skin fibroblasts to THz radiation with an electric field strength of 3.5 MV/cm for 30 min resulted in the growth of the number of H2AX foci in cells. Thus, the irradiation time of cell cultures of *t* = 0.5 h was chosen. The estimations performed earlier showed that the thermal treatment of the cell culture is minimal, and the temperature elevation at the spot of the THz treatment does not exceed 2.8 °C [62]. The size of the irradiated area of cells was determined by the size of the focused beam, i.e., ~480 µm at the 1/*e* level. To facilitate finding this area during the subsequent analysis of cells, its borders were laser-engraved. To do so, the THz crystal and filters were temporarily removed from the scheme, and the energy of laser pulses was attenuated using a polarization attenuator. The markers represented well-defined regions of the Petri dish material ablation in the corners of a 400 µm-sided square.

### 4.5. Introduction of a Click-iT EdU Label

To incorporate and detect EdU in DNA, we used a Click-iT Alexa Fluor 647 visualization kit (ThermoFisher Click-iT Plus EdU, Carlsbad, CA, USA). To incorporate the EdU in DNA, an earlier-prepared EdU solution in DMSO at the final concentration of 10 µM was added to the cell culture one day before the treatment.

### 4.6. Immunocytochemical Analysis

To perform the immunocytochemical analysis, cells were fixed in a Petri dish with a 4% solution of buffered formalin, containing 0.1% saponin, for 20 min at room temperature, with the following twofold washing with DPBS. Then, the cells were incubated for 1.5 h at 37 °C with anti-γH2AX (dilution of 1:1000, abcam11174), anti-β-III-tubulin (R&D, 2 g/mL), anti-GFAP (DAKO, 5 g/mL), anti-SOX2 (BD Biosciences, 5 g/mL), and anti-MAP2b (Sigma-Aldrich, St. Louis, MO, USA, 5 g/mL) rabbit primary polyclonal antibodies, preliminarily dissolved in DPBS with 0.5% Triton-X100 and 0.5% Tween 20, with the addition of 1% goat serum for blocking the non-specific binding of antibodies. After the incubation, the cells were washed thrice with DPBS containing 0.5% Triton-X100 and 0.5% Tween 20 and incubated for 1 h with secondary goat anti-rabbit IgG (H + L) antibodies (conjugated with Alexa Fluor 633 and Alexa Fluor 488, at a dilution of 1:400; Invitrogen, Waltham, MA, USA), also dissolved in DPBS with 0.5% Triton-X100 and 0.5% Tween 20, with the addition of 1% goat serum. Then, the Petri dishes were washed thrice with DPBS. The cell nuclei were stained with Hoechst 33342 (Thermo Fisher Scientific).

To detect the EdU, the cells were fixed with a 4% solution of buffered formalin, thrice washed with DPBS containing 0.5% Triton-X100 and 0.5% Tween 20, and incubated with the Click-iT Plus reaction cocktail (which included Alexa Fluor 633) for 1 h. Then, the Petri dishes were washed twice with DPBS, and the cells were stained for other antibodies in accordance with the above protocol.

The subsequent immunofluorescent study was performed using a CELENA^®^ S Digital Imaging System, v1.1.1 (Logos Biosystems, Anyang, Republic of Korea).

### 4.7. Statistical Analysis

The estimation of the number of foci of double-strand DNA breaks was performed based on the ratio of the γH2AX foci number to the number of cells in the S phase within the irradiation area. The experiment was repeated thrice. The statistical data were presented as the mean ± SD. The analysis of the statistical significance for the small samples was performed using Welch’s *t*-test. *T* was calculated according to the formula:

(1) $$T_{emp}=\frac{\sqrt{m\times n}\left|\bar{x}-\bar{y}\right|}{\sqrt{m\times S_{x}^{2}+n\times S_{y}^{2}}},$$

where $T_{emp}$ is the calculated *t* value; *m* and *n* are the sizes of the analyzed samples; $\bar{x}\;\mathrm{and}\;\bar{y}$ are the mean values for the samples *x* and *y*, respectively; $S_{x}^{2}$ and $S_{y}^{2}$ are the dispersion values for these samples. The obtained result was compared to the *T_cr_* = 1.96 for the given degrees of freedom (*p* < 0.05).

## 5. Conclusions

Using the technique for identification of cells in the S phase of the cell cycle, it was found that the proliferative activity of both non-tumor and tumor cells had not changed after the 30 min exposure to intense THz radiation (the intensity of 21 GW/cm^2^ and the electric field strength of 2.8 MV/cm) of the terahertz range. The increase in the number of the foci of histone H2AX phosphorylation was not statistically significant. Upon the combined treatment with salinomycin and THz radiation, the antibiotic played the most essential role; thus, the additive effect was almost absent. The estimation of the spontaneous differentiation of the neural progenitor cells (drNPCs) and the human neuroblastoma cells (SK-N-BE (2)) upon the 30 min exposure to radiation, based on the expression of the markers of β-III-tubulin, GFAP, SOX2, and the pro-neuronal MAP2b marker did not reveal any changes, though some decrease in the number of SOX2-positive and GFAP and β-III-tubulin co-expressing cells was observed, indicating the loss of the drNPC culture’s stem properties. The presence of MAP2b-positive cells with long sprouts indicated differentiation towards mature neurons. Thus, the results obtained suggest that THz pulses of given parameters induce neither a noticeable genotoxic effect on human neural progenitor cells (drNPCs) and neuroblastoma cells (SK-N-BE (2)) nor a statistically significant change in the proliferative activity and cell differentiation.

Terahertz radiation has significant potential in medical diagnosis and treatment due to its frequency range matching the characteristic energy of the biomolecular motion. A variety of cancer types have been detected by imaging and spectroscopy techniques. Huge prospects for treatment are associated with the demethylation of malignant DNA at specific THz frequencies. Further studies are required to find the optimal parameters of the THz exposure and to demonstrate the synergy with traditional chemotherapy and novel anti-tumor approaches.

## Data Availability

The data presented in this study are available on request from the corresponding author.

## References

[1] Zaytsev K.I., Dolganova I.N., Chernomyrdin N.V., Katyba G.M., Gavdush A.A., Cherkasova O.P., Komandin G.A., Shchedrina M.A., Khodan A.N., Ponomarev D.S. (2020). The Progress and Perspectives of Terahertz Technology for Diagnosis of Neoplasms: A Review. J. Opt..

[2] Zaytsev K.I., Kurlov V.N., Skorobogatiy M., Reshetov I.V., Tuchin V.V. (2021). Special Section Guest Editorial: Advances in Terahertz Biomedical Science and Applications. J. Biomed. Opt..

[3] Nikitkina A.I., Bikmulina P.Y., Gafarova E.R., Kosheleva N.V., Efremov Y.M., Bezrukov E.A., Butnaru D.V., Dolganova I.N., Chernomyrdin N.V., Cherkasova O.P. (2021). Terahertz Radiation and the Skin: A Review. J. Biomed. Opt..

[4] Son J.-H., Oh S.J., Cheon H. (2019). Potential Clinical Applications of Terahertz Radiation. J. Appl. Phys..

[5] Gavdush A.A., Chernomyrdin N.V., Komandin G.A., Dolganova I.N., Nikitin P.V., Musina G.R., Katyba G.M., Kucheryavenko A.S., Reshetov I.V., Potapov A.A. (2021). Terahertz Dielectric Spectroscopy of Human Brain Gliomas and Intact Tissues Ex Vivo: Double-Debye and Double-Overdamped-Oscillator Models of Dielectric Response. Biomed. Opt. Express.

[6] Chernomyrdin N.V., Musina G.R., Nikitin P.V., Dolganova I.N., Kucheryavenko A.S., Alekseeva A.I., Wang Y., Xu D., Shi Q., Tuchin V.V. (2023). Terahertz Technology in Intraoperative Neurodiagnostics: A Review. Opto-Electron. Adv..

[7] Musina G.R., Chernomyrdin N.V., Gafarova E.R., Gavdush A.A., Shpichka A.J., Komandin G.A., Anzin V.B., Grebenik E.A., Kravchik M.V., Istranova E.V. (2021). Moisture Adsorption by Decellularized Bovine Pericardium Collagen Matrices Studied by Terahertz Pulsed Spectroscopy and Solid Immersion Microscopy. Biomed. Opt. Express.

[8] Titova L.V., Ayesheshim A.K., Golubov A., Rodriguez-Juarez R., Woycicki R., Hegmann F.A., Kovalchuk O. (2013). Intense THz Pulses Down-Regulate Genes Associated with Skin Cancer and Psoriasis: A New Therapeutic Avenue?. Sci. Rep..

[9] Serdyukov D.S., Goryachkovskaya T.N., Mescheryakova I.A., Kuznetsov S.A., Popik V.M., Peltek S.E. (2021). Fluorescent Bacterial Biosensor E Coli/PTdcR-TurboYFP Sensitive to Terahertz Radiation. Biomed. Opt. Express.

[10] Wilmink G.J., Grundt J.E. (2011). Invited Review Article: Current State of Research on Biological Effects of Terahertz Radiation. J. Infrared Millim. Terahertz Waves.

[11] Il’ina I.V., Sitnikov D.S., Agranat M.B. (2018). State-of-the-Art of Studies of the Effect of Terahertz Radiation on Living Biological Systems. High Temp..

[12] Cherkasova O.P., Serdyukov D.S., Ratushnyak A.S., Nemova E.F., Kozlov E.N., Shidlovskii Y.V., Zaytsev K.I., Tuchin V.V. (2020). Effects of Terahertz Radiation on Living Cells: A Review. Opt. Spectrosc..

[13] Cherkasova O.P., Serdyukov D.S., Nemova E.F., Ratushnyak A.S., Kucheryavenko A.S., Dolganova I.N., Xu G., Skorobogatiy M., Reshetov I.V., Timashev P.S. (2021). Cellular Effects of Terahertz Waves. J. Biomed. Opt..

[14] Romeo S., Zeni O., Scarfì M., Poeta L., Lioi M., Sannino A. (2022). Radiofrequency Electromagnetic Field Exposure and Apoptosis: A Scoping Review of In Vitro Studies on Mammalian Cells. Int. J. Mol. Sci..

[15] Stefi A.L., Margaritis L.H., Skouroliakou A.S., Vassilacopoulou D. (2019). Mobile Phone Electromagnetic Radiation Affects Amyloid Precursor Protein and α-Synuclein Metabolism in SH-SY5Y Cells. Pathophysiology.

[16] Zhi W.-J., Wang L.-F., Hu X.-J. (2017). Recent Advances in the Effects of Microwave Radiation on Brains. Mil. Med. Res..

[17] Zhao X., Zhang M., Liu Y., Liu H., Ren K., Xue Q., Zhang H., Zhi N., Wang W., Wu S. (2021). Terahertz Exposure Enhances Neuronal Synaptic Transmission and Oligodendrocyte Differentiation in Vitro. iScience.

[18] Tan S.Z., Tan P.C., Luo L.Q., Chi Y.L., Yang Z., Zhao X., Zhao L., Dong J., Zhang J., Yao B.W. (2019). Exposure Effects of Terahertz Waves on Primary Neurons and Neuron-like Cells Under Nonthermal Conditions. Biomed. Environ. Sci..

[19] Romanenko S., Begley R., Harvey A.R., Hool L., Wallace V.P. (2017). The Interaction between Electromagnetic Fields at Megahertz, Gigahertz and Terahertz Frequencies with Cells, Tissues and Organisms: Risks and Potential. J. R. Soc. Interface.

[20] Alexandrov B.S., Lisa Phipps M., Alexandrov L.B., Booshehri L.G., Erat A., Zabolotny J., Mielke C.H., Chen H.T., Rodriguez G., Rasmussen K.O. (2013). Specificity and Heterogeneity of Terahertz Radiation Effect on Gene Expression in Mouse Mesenchymal Stem Cells. Sci. Rep..

[21] Titova L.V., Ayesheshim A.K., Golubov A., Fogen D., Rodriguez-Juarez R., Hegmann F.A., Kovalchuk O. (2013). Intense THz Pulses Cause H2AX Phosphorylation and Activate DNA Damage Response in Human Skin Tissue. Biomed. Opt. Express.

[22] Sitnikov D.S., Ilina I.V., Gurova S.A., Shatalova R.O., Revkova V.A. (2020). Studying the Induction of Double-Strand Breaks in Human Fibroblasts by High-Intensity Terahertz Radiation. Bull. Russ. Acad. Sci. Phys..

[23] Sitnikov D.S., Ilina I.V., Revkova V.A., Rodionov S., Gurova S., Shatalova R., Kovalev A., Ovchinnikov A.V., Chefonov O.V., Konoplyannikov M. (2021). Effects of High Intensity Non-Ionizing Terahertz Radiation on Human Skin Fibroblasts. Biomed. Opt. Express.

[24] Wollner I.S., Prust R.M., Andrews J.C., Walker-Andrews S.C., Nostrant T.T., Knol J.A., Eckhauser F.E., Cho K.J., Lichter A.S., Ensminger W.D. (1989). Combination Chemo-Radiation Therapy for Jaundice Due to Focal Malignant Obstruction of the Major Bile Ducts. Sel. Cancer Ther..

[25] Wagner T., Yang G. (2008). Cetuximab: Its Use in Combination with Radiation Therapy and Chemotherapy in the Multimodality Treatment of Head and Neck Cancer. Recent Pat. Anticancer. Drug Discov..

[26] Gupta P.B., Onder T.T., Jiang G., Tao K., Kuperwasser C., Weinberg R.A., Lander E.S. (2009). Identification of Selective Inhibitors of Cancer Stem Cells by High-Throughput Screening. Cell.

[27] Fuchs D., Heinold A., Opelz G., Daniel V., Naujokat C. (2009). Salinomycin Induces Apoptosis and Overcomes Apoptosis Resistance in Human Cancer Cells. Biochem. Biophys. Res. Commun..

[28] Zhang G.-N., Liang Y., Zhou L.-J., Chen S.-P., Chen G., Zhang T.-P., Kang T., Zhao Y.-P. (2011). Combination of Salinomycin and Gemcitabine Eliminates Pancreatic Cancer Cells. Cancer Lett..

[29] Dong T.-T., Zhou H.-M., Wang L.-L., Feng B., Lv B., Zheng M.-H. (2011). Salinomycin Selectively Targets ‘CD133+’ Cell Subpopulations and Decreases Malignant Traits in Colorectal Cancer Lines. Ann. Surg. Oncol..

[30] Lu D., Choi M.Y., Yu J., Castro J.E., Kipps T.J., Carson D.A. (2011). Salinomycin Inhibits Wnt Signaling and Selectively Induces Apoptosis in Chronic Lymphocytic Leukemia Cells. Proc. Natl. Acad. Sci. USA.

[31] Norouzi M., Yathindranath V., Thliveris J.A., Miller D.W. (2020). Salinomycin-Loaded Iron Oxide Nanoparticles for Glioblastoma Therapy. Nanomaterials.

[32] Zhou J., Liu S., Wang Y., Dai W., Zou H., Wang S., Zhang J., Pan J. (2019). Salinomycin Effectively Eliminates Cancer Stem-like Cells and Obviates Hepatic Metastasis in Uveal Melanoma. Mol. Cancer.

[33] Wang F., Zhou S., Qi D., Xiang S.-H., Wong E.T., Wang X., Fonkem E., Hsieh T., Yang J., Kirmani B. (2019). Nucleolin Is a Functional Binding Protein for Salinomycin in Neuroblastoma Stem Cells. J. Am. Chem. Soc..

[34] Cheon H., Paik J.H., Choi M., Yang H.J., Son J.H. (2019). Detection and Manipulation of Methylation in Blood Cancer DNA Using Terahertz Radiation. Sci. Rep..

[35] Baklaushev V.P., Bogush V.G., Kalsin V.A., Sovetnikov N.N., Samoilova E.M., Revkova V.A., Sidoruk K.V., Konoplyannikov M.A., Timashev P.S., Kotova S.L. (2019). Tissue Engineered Neural Constructs Composed of Neural Precursor Cells, Recombinant Spidroin and PRP for Neural Tissue Regeneration. Sci. Rep..

[36] Revkova V.A., Sidoruk K.V., Kalsin V.A., Melnikov P.A., Konoplyannikov M.A., Kotova S., Frolova A.A., Rodionov S.A., Smorchkov M.M., Kovalev A.V. (2021). Spidroin Silk Fibers with Bioactive Motifs of Extracellular Proteins for Neural Tissue Engineering. ACS Omega.

[37] Sedelnikova O.A., Pilch D.R., Redon C., Bonner W.M. (2003). Histone H2AX in DNA Damage and Repair. Cancer Biol. Ther..

[38] Kinner A., Wu W., Staudt C., Iliakis G. (2008). γ-H2AX in Recognition and Signaling of DNA Double-Strand Breaks in the Context of Chromatin. Nucleic Acids Res..

[39] Dhuppar S., Roy S., Mazumder A. (2020). ΓH2AX in the S Phase after UV Irradiation Corresponds to DNA Replication and Does Not Report on the Extent of DNA Damage. Mol. Cell. Biol..

[40] Konoplyannikov M.A., Eremina A.S., Kargina Y.V., Le-Deygen I.M., Kharin A.Y., Bazylenko T.Y., Yusubalieva G.M., Revkova V.A., Matchuk O.N., Zamulaeva I.A. (2021). Mesoporous Silicon Nanoparticles Loaded with Salinomycin for Cancer Therapy Applications. Microporous Mesoporous Mater..

[41] Clothier R.H., Bourne N. (2003). Effects of THz Exposure on Human Primary Keratinocyte Differentiation and Viability. J. Biol. Phys..

[42] Bourne N., Clothier R.H., D’Arienzo M., Harrison P. (2008). The Effects of Terahertz Radiation on Human Keratinocyte Primary Cultures and Neural Cell Cultures. ATLA Altern. Lab. Anim..

[43] Yu T., MacPhail S.H., Banáth J.P., Klokov D., Olive P.L. (2006). Endogenous Expression of Phosphorylated Histone H2AX in Tumors in Relation to DNA Double-Strand Breaks and Genomic Instability. DNA Repair.

[44] Bogomazova A.N., Vassina E.M., Goryachkovskaya T.N., Popik V.M., Sokolov A.S., Kolchanov N.A., Lagarkova M.A., Kiselev S.L., Peltek S.E. (2015). No DNA Damage Response and Negligible Genome-Wide Transcriptional Changes in Human Embryonic Stem Cells Exposed to Terahertz Radiation. Sci. Rep..

[45] Sitnikov D.S., Revkova V.A., Ilina I.V., Gurova S.A., Komarov P.S., Struleva E.V., Konoplyannikov M.A., Kalsin V.A., Baklaushev V.P. (2023). Studying the Genotoxic Effects of High Intensity Terahertz Radiation on Fibroblasts and CNS Tumor Cells. J. Biophotonics.

[46] Hintzsche H., Jastrow C., Kleine-Ostmann T., Kärst U., Schrader T., Stopper H. (2012). Terahertz Electromagnetic Fields (0.106 THz) Do Not Induce Manifest Genomic Damage In Vitro. PLoS ONE.

[47] Moskaleva E.Y., Severin S.E. (2012). Antitumor Activity of Ionophore Antibiotic Salinomycin: The Target—Cancer Stem Cells. Mol. Med..

[48] Mai T.T., Hamaï A., Hienzsch A., Cañeque T., Müller S., Wicinski J., Cabaud O., Leroy C., David A., Acevedo V. (2017). Salinomycin Kills Cancer Stem Cells by Sequestering Iron in Lysosomes. Nat. Chem..

[49] Liu Q., Sun J., Luo Q., Ju Y., Song G. (2021). Salinomycin Suppresses Tumorigenicity of Liver Cancer Stem Cells and Wnt/Beta-Catenin Signaling. Curr. Stem Cell Res. Ther..

[50] Naujokat C., Steinhart R. (2012). Salinomycin as a Drug for Targeting Human Cancer Stem Cells. J. Biomed. Biotechnol..

[51] Liu Y., Hao Y., Li Y., Zheng Y., Dai J., Zhong F., Wei W., Fang Z. (2020). Salinomycin Induces Autophagic Cell Death in Salinomycin-Sensitive Melanoma Cells through Inhibition of Autophagic Flux. Sci. Rep..

[52] Amini T., Jahangiri F., Ameri Z., Hemmatian M.A. (2021). A Review of Feasible Applications of THz Waves in Medical Diagnostics and Treatments. J. Lasers Med. Sci..

[53] Vicario C., Ovchinnikov A.V., Ashitkov S.I., Agranat M.B., Fortov V.E., Hauri C.P. (2014). Generation of 09-MJ THz Pulses in DSTMS Pumped by a Cr:Mg_2SiO_4 Laser. Opt. Lett..

[54] Sitnikov D.S., Ilina I.V., Pronkin A.A. (2020). Experimental System for Studying Bioeffects of Intense Terahertz Pulses with Electric Field Strength up to 3.5 MV/cm. Opt. Eng..

[55] Sitnikov D.S., Ilina I.V., Revkova V.A., Konoplyannikov M.A., Kalsin V.A., Baklaushev V.P. (2020). System for Long-Term Irradiation of Living Cell Culture with High-Intensity THz Pulses. High Temp..

[56] Ovchinnikov A.V., Chefonov O.V., Sitnikov D.S., Il’ina I.V., Ashitkov S.I., Agranat M.B. (2018). A Source of THz Radiation with Electric Field Strength of More than 1 MV Cm -1 on the Basis of 100-Hz Femtosecond Cr: Forsterite Laser System. Quantum Electron..

[57] Vicario C., Jazbinsek M., Ovchinnikov A.V., Chefonov O.V., Ashitkov S.I., Agranat M.B., Hauri C.P. (2015). High Efficiency THz Generation in DSTMS, DAST and OH1 Pumped by Cr:Forsterite Laser. Opt. Express.

[58] Nazarov M.M., Shcheglov P.A., Teplyakov V.V., Chashchin M.V., Mitrofanov A.V., Sidorov-Biryukov D.A., Panchenko V.Y., Zheltikov A.M. (2021). Broadband Terahertz Generation by Optical Rectification of Ultrashort Multiterawatt Laser Pulses near the Beam Breakup Threshold. Opt. Lett..

[59] Ovchinnikov A.V., Chefonov O.V., Agranat M.B., Shalaby M., Sitnikov D.S. (2022). Terahertz Generation Optimization in an OH1 Nonlinear Organic Crystal Pumped by a Cr:Forsterite Laser. Opt. Lett..

[60] Sitnikov D.S., Romashevskiy S.A., Ovchinnikov A.V., Chefonov O.V., Savel’ev A.B., Agranat M.B. (2019). Estimation of THz Field Strength by an Electro-Optic Sampling Technique Using Arbitrary Long Gating Pulses. Laser Phys. Lett..

[61] Ahlfors J.-E., Azimi A., El-Ayoubi R., Velumian A., Vonderwalde I., Boscher C., Mihai O., Mani S., Samoilova M., Khazaei M. (2019). Examining the Fundamental Biology of a Novel Population of Directly Reprogrammed Human Neural Precursor Cells. Stem Cell Res. Ther..

[62] Sitnikov D.S., Pronkin A.A., Ilina I.V., Revkova V.A., Konoplyannikov M.A., Kalsin V.A., Baklaushev V.P. (2021). Numerical Modelling and Experimental Verification of Thermal Effects in Living Cells Exposed to High-Power Pulses of THz Radiation. Sci. Rep..

